# Variability in sensitivity to inflammation in muscle and lung of patients with COPD may underlie susceptibility to lung function decline

**DOI:** 10.1136/thorax-2024-221901

**Published:** 2025-04-16

**Authors:** Paul R Kemp, Mark Griffiths, Michael I Polkey, Amanda Sathyapala

**Affiliations:** 1 National Heart and Lung Institue, Imperial College London, London, UK; 2 Barts Heart Centre, St Bartholomew’s Hospital, London, UK; 3 NIHR Respiratory BRU, Guy's and St Thomas’ Hospitals NHS Trust, London, UK; 4 Guy's and St Thomas’ NHS Foundation Trust, London, UK

**Keywords:** COPD Pathology, Systemic disease and lungs, Cytokine Biology

## Abstract

**Background:**

Muscle wasting and weakness (sarcopenia) are commonly associated with COPD causing frailty and reduced quality of life. The contribution of inflammation to muscle loss and the susceptibility to rapid lung function decline is debated. We hypothesised that comparing the muscle transcriptome to circulating inflammatory cytokine profiles in patients would identify any contribution of systemic inflammation to muscle atrophy.

**Methods:**

Quadriceps differential gene expression was determined between mild-COPD (n=28) and severe-COPD (n=51) using GSE100281. These microarray data were compared by biweight mid-correlation with lung function and plasma cytokine levels from the same patients.

**Results:**

Patients with severe COPD had reduced fat-free mass index (a measurement of muscle mass) compared with patients with mild COPD despite similar physical activity and inflammatory cytokine levels. Gene sets associated with inflammation and epithelial mesenchymal transition (EMT) were elevated in severe COPD, suggesting that inflammation may contribute to the loss of muscle mass. In patients with severe COPD, EMT and inflammation gene sets were strongly associated with circulating proinflammatory and anti-inflammatory cytokines. However, in patients with mild COPD, anti-inflammatory cytokines showed negative associations with these gene sets and associations with proinflammatory cytokines were weak. In data from lung and blood samples, patients with severe COPD had elevated inflammatory and EMT gene expression compared with patients with mild COPD suggesting that this phenomenon is not muscle-specific.

**Conclusions:**

In patients at the severe end of the COPD spectrum, the proinflammatory response in muscle predominates, whereas in patients at the mild end of the spectrum, the anti-inflammatory response predominates. This suggestion needs confirming in a longitudinal cohort.

**WHAT IS ALREADY KNOWN ON THIS TOPIC**

- Inflammation plays a key role in the pathogenesis of COPD and sarcopenia associated with various diseases. However, the role that individual susceptibility to inflammation plays in COPD disease progression and associated sarcopenia is poorly understood.

**WHAT THIS STUDY ADDS**

- Correlation of the transcriptomes of muscle and lung in patients with mild and severe COPD with markers of bloodborne inflammation suggests that patients with mild COPD may be relatively protected by an anti-inflammatory profile and patients with severe COPD are relatively susceptible to circulating proinflammatory mediators.

**HOW THIS STUDY MIGHT AFFECT RESEARCH, PRACTICE OR POLICY**

- This study suggests that it may be possible to select susceptible patients for early anti-inflammatory intervention to prevent muscle atrophy and progressive COPD. Longitudinal studies and further investigation of individual pathways are required to validate our findings.

## Introduction

 Muscle loss limits the functional capacity of patients with Chronic Obstructive Pulmonary disease (COPD) and other chronic diseases, reducing their ability to perform the tasks of daily living, their quality of life and lifespan.^1^

Chronic inflammation is common to diseases that lead to muscle wasting,^2^ including COPD. Inflammation increases protein turnover, increasing blood urea nitrogen, leading to a negative nitrogen balance,^3^ and inflammatory cytokines promote protein breakdown in both in vitro and in vivo studies.^4^,^6^ While inflammation is likely to play a key role in COPD-associated muscle loss, some studies show associations of circulating inflammatory cytokines with muscle loss, whereas others do not.^7 8^ There are several potential reasons for this variability. First, inflammation is not the only proposed mechanism of muscle loss, with inactivity, oxidative stress and malnutrition all proposed as important regulators.^9^ Second, there is significant interplay between these mechanisms, with inflammatory cytokines reducing appetite and activity, altering mood and motivation, and increasing oxidative stress.^10^ Each of these responses adds variability to the data, with different individuals having different changes in activity, for example, for a given inflammatory load. Third, there are multiple potential inflammatory mediators which can contribute to the loss of muscle mass, making it difficult to identify the effects of individual cytokines. Fourthly, each factor (inflammation, inactivity, etc) may have greater relative importance at different stages of the disease. Lastly, some cytokine components of the inflammatory response have anti-inflammatory activity,^11^ so the relative levels of proinflammatory and antiinflammatory cytokines determine the responses of target tissues.

Furthermore, individuals are likely to have different intrinsic sensitivities to inflammatory stimulus in the muscle and other tissues.^12 13^ Mechanisms that could affect sensitivity include different levels of expression of receptors or signalling intermediates that affect the signal provided by a given amount of cytokine. Such variation is likely to be both genetically and environmentally determined. Consistent with such a suggestion, analyses of individual polymorphisms and a meta-analysis of Genome Wide Association Study (GWAS) data have implicated toll-like receptors^14^,^16^ as well as interleukins and their receptors^17^,^19^ as susceptibility loci for lung function and rate of lung function decline. A meta-analysis of GWAS data also implicated transcription factors including NF-Kb1, signal transducer and activator of transcription (STAT) 1, 2 and 6, KLF4, 12 and 16 that are involved in inflammatory responses.^12^

The changes in protein turnover and gene expression that cause muscle loss are likely to reflect a co-ordinated programme of responses to the circulating milieu. We hypothesised that the altered programme may be elucidated by comparing levels of circulating cytokines with the skeletal muscle transcriptome, thereby getting a more detailed picture of the changes in gene expression that any individual cytokine is responsible for. We have previously measured a panel of inflammatory cytokines in the plasma of well-phenotyped patients with COPD^20^ and separately quantified the skeletal muscle transcriptome by microarray in the same patients.^21^ However, we have not previously combined these data sets. We, therefore, compared plasma cytokine levels with the quadriceps transcriptome to determine the relative importance of each cytokine to skeletal muscle phenotype. Subsequently, we obtained published transcriptome data from studies of lung and blood in patients with COPD, where relative disease severity or lung function data were also available, and compared gene expression profiles within these data sets for validation.^22 23^

## Methods

### Patients

The subjects in this study are subsets from a previously reported cohort of patients with COPD.^24^ The inclusion and exclusion criteria and descriptions of the physiological analysis have been described previously.^24^ Biopsies of the vastus lateralis were taken by Bergstrom needle and plasma prepared from whole blood at the same visit. The methodology for the assessment of all clinical and lung function parameters (forced expiratory volume in 1 s and capacity of the lung for the transfer of carbon monoxide (FEV_1_ and TL_CO_)), exercise capacity (6 min walk distance) as well as physical activity (time spent moving) is described in the previous publications.^24^,^26^ The demographics of the whole cohort and for the microarray cohort are given in online supplemental tables S1 and S2, respectively.

Inflammatory cytokines and CRP were quantified in plasma samples as previously described.^20^ The methodology and initial analysis of muscle fibre type has been described,^24^ as has the generation and overall analysis of the RNA isolated from the same patients.^21^

### Statistical analysis

Patients were divided into two groups based on Global Initiative for Chronic Obstructive Lung Disease (GOLD) criteria: GOLD 1 or 2 as mild and GOLD 3 or 4 as severe COPD.

Normality of variables within each group was determined by the Shapiro-Wilk test. Differences in phenotypic variables across the three groups (control, mild and severe) were determined by analysis of variance in Aabel 3.0 for normally distributed variables using the Dunn-Bonferroni correction for multiple testing. For phenotypic variables that did not show a normal distribution, these groups were compared using the Kruskal-Wallis test in SPSS V.24 with a Dunn-Bonferroni correction.

### Bioinformatic analysis

The muscle microarray data were generated using Affymetrix Human Gene 1.1 ST microarray (covering 20 926 different genes). The data have been published^21^ and are available as GSE100281. Data for all 79 patients with COPD were used in this study (28 mild COPD and 51 severe COPD). Differences in gene expression between groups were determined using the R package LIMMA (Linear Models for Microarray Data) and the output loaded into GSEA (Gene Set Enrichment Analysis) using fold difference as the ranking metric. Significance of gene set enrichment is presented as false discovery rate calculated within the GSEA programme to account for gene set size as described.^27^ To identify differentially enriched gene sets, the rank order was compared with the Hallmark gene sets.

To identify gene associations with circulating cytokines and TL_CO_ % predicted, gene expression data for the relevant patients was compared with cytokine levels using biweight mid correlation in the R package Weighted Genome Co-expression Network Analysis. The correlation coefficient was then used as the ranking metric in GSEA again using comparison with the Hallmark gene sets (see online supplemental methods).

Lung biopsy and whole blood RNA data were downloaded from previously published datasets (GSE69818^22^ and GSE71220^23^, respectively). GSE69818 is a microarray study of lung tissue from patients requiring transplantation or cancer resection, and for our study, it was analysed by including all samples (n=70, 51 mild and 19 severe) and by selecting only those with emphysema to remove potential bias (n=38, 22 mild and 16 severe). The array was run using the Affymetrix GeneChip Human Genome U219 of 19 943 genes. GSE71220 is a study of whole blood from patients with COPD comparing the effect of statins on gene expression profile. Gene expression was profiled using the Affymetrix Human Gene 1.1 ST microarray covering 20 926 different genes. Only those not taking statins were selected (n=451) to remove any effect of the drug from the result. Each data set was divided into mild and severe COPD based on GOLD grade and analysed using LIMMA and GSEA as described above.

## Results

**Figure 1:**
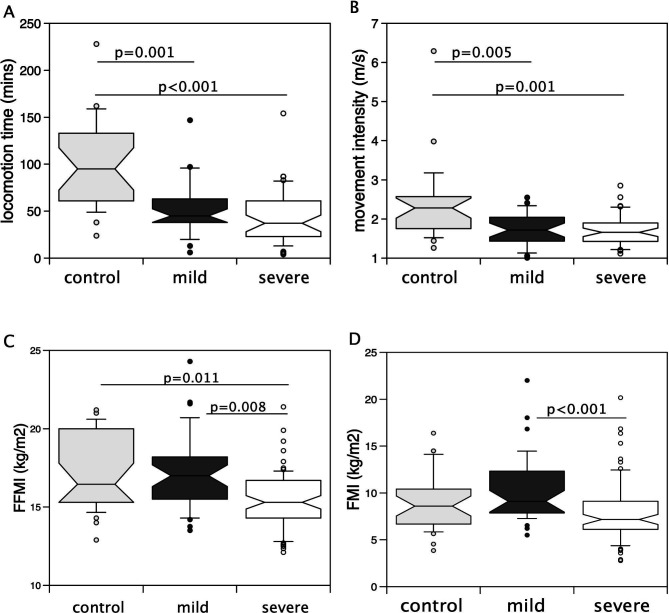
Comparison of activity and fat-free mass index (FFMI) and fat mass index (FMI) with severity of Chronic Obstructive Pulmonary disease (COPD). Locomotion time (**A**) and the intensity of movement (**B**) determined by triaxial accelerometery were lower in both patient groups compared with controls but did not differ between patients with mild and severe COPD. Conversely, FFMI (**C**) was different between the severe patients compared with the other two groups but not between mild patients and controls. FMI (**D**) was lower in severe patients than the mild patients but there was no difference between either patient group and controls.

Comparisons of muscle gene expression between patients with COPD and controls may be complicated by differences in physical activity. Comparison of activity between healthy controls, mild COPD and severe COPD groups showed that both patient groups had reduced locomotion time and movement intensity, but there was no difference in these measures of activity between the two patient groups (figure 1A,B). Comparison of body composition of these three groups showed that patients with mild COPD had similar fat-free mass index (FFMI) to controls, but a higher FFMI than patients with severe COPD. They also had a higher fat mass index (FMI) than patients with severe COPD (figure 1C,D). Consequently, comparing gene expression profiles between mild and severe COPD will identify differences in muscle gene expression associated with FFMI in the absence of the effects of activity.

Differential gene expression analysis in muscle biopsies followed by GSEA showed increased expression of gene sets associated with the epithelial mesenchymal transition (EMT) and inflammation (tumour necrosis factor alpha (TNF-α) signalling) in severe COPD compared with mild COPD (table 1), an observation supported by GSEA following correlation of the transcriptome with TLCO_%pred_ (online supplemental table S3). The enrichment for inflammatory gene sets occurred without a difference in circulating inflammatory cytokine levels between mild and severe patients (online supplemental table S4A, B). Consequently, the circulating inflammatory milieu did not account for increased inflammatory signalling in the muscle of patients with severe COPD.

**Table 1:** Gene set enrichment for differentially expressed genes in the quadriceps of patients with severe and those with mild COPD

Hallmark gene set	NES	FDR q-val
Positive enrichment		
EMT	2.28	<0.001
TNFA signalling via NFKB	1.81	0.007
Heme metabolism	1.78	0.007
UV response down	1.72	0.008
Apical junction	1.64	0.016
Angiogenesis	1.61	0.021
Negative enrichment		
Fatty acid metabolism	−2.40	<0.001
Oxidative phosphorylation	−2.08	0.002
Peroxisome	−1.72	0.035

Differential gene expression was determined using LIMMA (severe–mild) and used to rank genes. The ranked genes were then analysed by GSEA using the Hallmark gene sets.

COPD, Chronic Obstructive Pulmonary disease; EMT, epithelial mesenchymal transition; FDR, false discovery rate; GSEA, Gene Set Enrichment Analysis; LIMMA, Linear Models for Microarray Data; NES, Normalised Enrichment Score; UV, Ultra-Violet.

To identify mechanisms contributing to the differences in muscle inflammatory response between patients with mild and severe COPD, we correlated cytokine levels with gene expression measured by array. In patients with severe COPD, for both proinflammatory and anti-inflammatory cytokines (table 2 and online supplemental tables S5–S11), genes ranked by correlation coefficient with the cytokine showed positive enrichment for the EMT and inflammatory gene sets (TNF-α signalling, interferon signalling and IL6 signalling). Conversely, in patients with mild COPD, the proinflammatory cytokines showed no or a weak positive enrichment for inflammatory gene sets with a reduced net enrichment score (NES) and at lower statistical significance compared with that in patients with severe COPD (table 2 and online supplemental tables S5–S11), whereas the anti-inflammatory cytokines showed negative enrichment for inflammatory gene sets (table 2). Although gender distribution did not differ between the groups, to ensure that there were no effects of differences between males and females, the analysis was repeated within males alone (as this group was large enough). This showed similar results (online supplemental results and online supplemental figure S1 and online supplemental tables S12–S16) with one addition where mild patients had higher fat mass than severe patients and controls, perhaps suggesting that in these individuals, the relative inactivity promoted fat accumulation. These data suggest that in patients with mild COPD, the anti-inflammatory response is sufficient to suppress muscle inflammatory signalling, whereas in severe COPD, the proinflammatory signal predominates.

**Table 2:** Gene set enrichment for genes expressed in the quadriceps correlating with circulating IL1B and IL10 in COPD

Mild COPD			Severe COPD		
Hallmark gene set	NES	FDR	Hallmark gene set	NES	FDR
**Positive enrichment with IL1B**					
EMT	1.72	0.029	EMT	3.14	<0.001
Interferon alpha response	1.68	0.026	Allograft rejection	3.06	<0.001
			Interferon gamma response	2.84	<0.001
			IL6 JAK STAT3 Signalling	2.70	<0.001
			TNFA signalling via NFKB	2.67	<0.001
			Inflammatory response	2.57	<0.001
			KRAS signalling up	2.50	<0.001
			Complement	2.47	<0.001
			Apoptosis	2.38	<0.001
			Interferon alpha response	2.37	<0.001
**Negative enrichment with IL1b**					
MTORC1 signalling	−1.94	0.004	Nothing		
Myc targets v1	−1.90	0.002			
Oxidative phosphorylation	−1.90	0.001			
Protein secretion	−1.76	0.005			
E2F targets	−1.75	0.005			
Unfolded protein response	−1.58	0.020			
Mitotic spindle	−1.51	0.033			
Myc targets v2	−1.50	0.032			
**Positive enrichment with IL10**					
Nothing			EMT	2.34	<0.001
			TNFA signalling via NFKB	2.30	<0.001
			IL6 JAK STAT3 signalling	2.20	<0.001
			Interferon gamma response	2.07	<0.001
			MTORC1 signalling	2.06	<0.001
			Myc targets v1	1.99	0.001
			Apoptosis	1.89	0.001
			Interferon alpha response	1.87	0.001
			Protein secretion	1.86	0.002
			Myc targets v2	1.85	0.001
**Negative enrichment with IL10**					
E2F targets	−2.22	<0.001	Oxidative phosphorylation	−2.51	<0.001
EMT	−2.20	<0.001	Fatty acid metabolism	−2.35	<0.001
Unfolded protein response	−2.09	<0.001	Bile acid metabolism	−1.98	0.000
TNFA signalling via NFkB	−2.08	<0.001	Wnt beta catenin signalling	−1.76	0.003
Interferon alpha response	−2.08	<0.001	Adipogenesis	−1.72	0.003
Mitotic spindle	−2.06	<0.001	Peroxisome	−1.46	0.037
Interferon gamma response	−2.05	<0.001			
Apoptosis	−2.04	<0.001			
G2M checkpoint	−1.92	<0.001			
Protein secretion	−1.91	<0.001			

Gene expression was correlated with IL1B or IL10 levels in circulation by robust biweight mid correlation and the genes ranked as described in online supplemental methods. The ranks were then analysed in GSEA.

COPD, Chronic Obstructive Pulmonary disease; EMT, epithelial mesenchymal transition; FDR, false discovery rate; GSEA, Gene Set Enrichment Analysis; NES, Normalised Enrichment Score.

**Figure 2:**
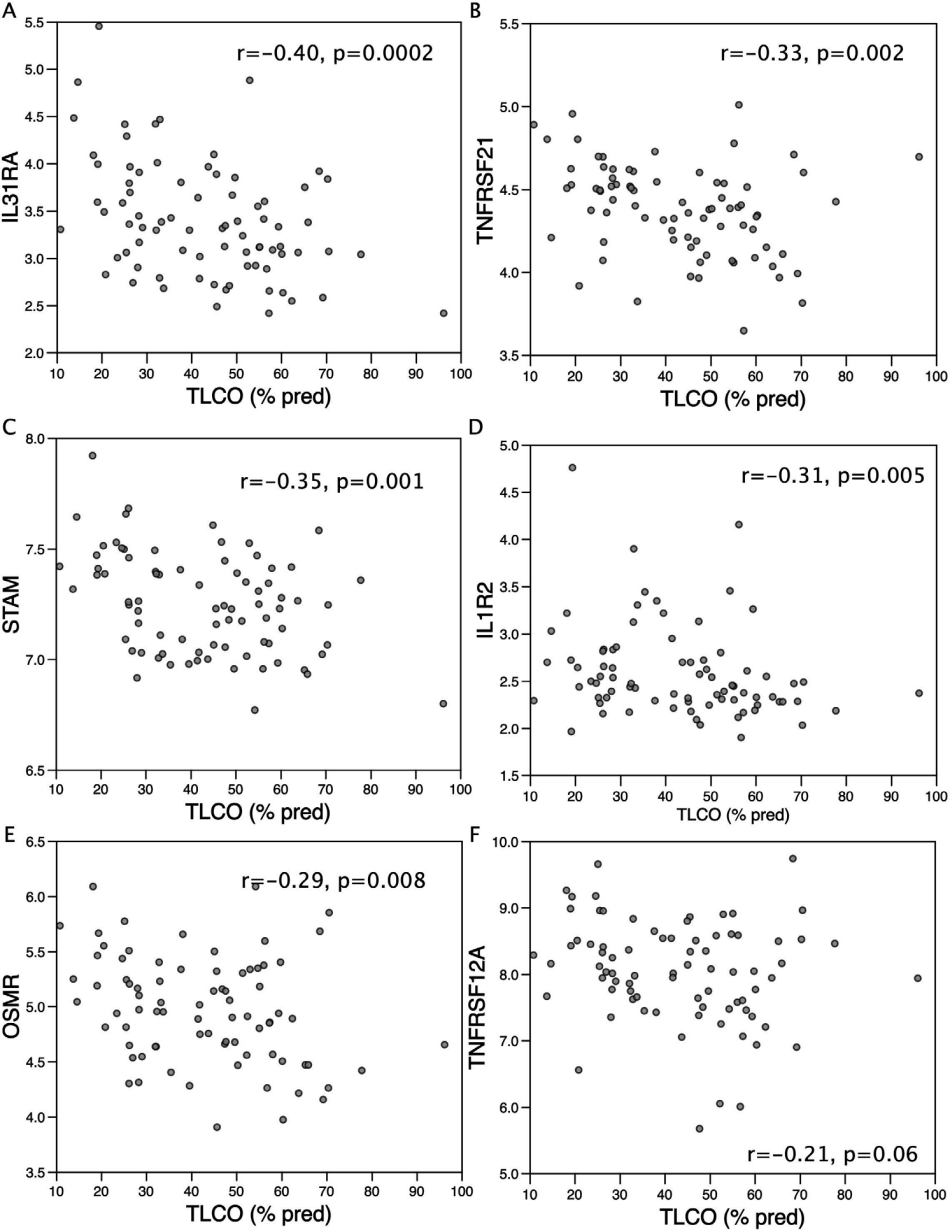
Expression of inflammatory receptors and signalling proteins in the muscle of patients with Chronic Obstructive Pulmonary disease (COPD). Expression (determined by microarray) of components of the inflammatory system identified as part of the IL6/JAK/STAT hallmark set and of IL31RA was compared with lung function (TL_CO_% predicted) in the patients. The expression of the IL31 receptor, IL31RA (**A**) TNF receptor superfamily member 21 TNFRSF21 (**B**), signal transducer adaptor molecule, STAM (**C**), IL1 receptor 2, IL1R2 (**D**), Oncostatin M receptor, OSMR (**E**), TNF receptor superfamily member 12A, TNFRSF12A (**F**).

The lack of difference in circulating cytokines between patients with mild and severe COPD suggests that severe patients have a greater response to circulating proinflammatory cytokines than patients with mild COPD or a weaker response to anti-inflammatory cytokines. Analysis of the inflammatory gene set with the greatest positive enrichment with TLCO_%pred_ (IL6/JAK/STAT3 signalling) showed that the strongest associations of individual genes with TLCO_%pred_ included inflammatory cytokine receptors (TNFRSF21, IL1R2, oncostatin M receptor (OSMR), TNSFR12A) and signal transducer/adaptor proteins (IRF1, signal transducing adaptor molecule 2 (STAM2), STAT1 and STAT2) (onlinesupplemental figure S2  table S17). Further analysis showed that the IL31 receptor (IL31RA), a coreceptor with OSMR, also associated with TL_CO_% pred but was not annotated in the IL6/JAK/STAT gene set (figure 2). Consistent with a role for inflammatory signalling in muscle wasting, IL31RA expression and STAM (a protein involved in cytokine receptor signalling) were inversely proportional to FFMI (online supplemental figure S2).

It is possible that increasing disease severity promotes the expression of inflammatory gene sets or that differences in gene expression cause individuals to progress through disease at different rates. As patients with severe disease must have had mild disease at some point, those with severe disease should be older than those with mild disease for a given level of smoking. However, despite similar smoking histories, patients with mild disease were older than those with severe disease, suggesting that the mild disease group was enriched for patients more resistant to the initiation or progression of the disease (online supplemental tables S1 and S2).

As the transcription factor MYC promotes EMT^28^ and MYC targets were also positively enriched in association with TLCO_%pred_, we compared MYC expression with the rest of the transcriptome. Positively enriched gene sets included the EMT, MYC targets and the inflammatory gene sets for TNF-α signalling, IL2 and IL6 signalling as well as interferon signalling (online supplemental table S18). MYC expression was directly associated with IL31RA, OSMR expression as well as with MCP-1 and IL6 expression (online supplemental figure S3).

We next examined published data sets for lung and blood from patients with COPD, where we could separate the patients into mild and severe disease. Differential gene expression analysis of lung biopsies (GSE69818) again showed enrichment for inflammatory gene expression gene sets, the EMT and MYC (table 3). This analysis was repeated using data only from those with emphysema, and the same results were obtained (online supplemental table S19). A similar differential gene expression analysis of RNA isolated from the blood of patients not receiving statins taken from GSE71220 also showed enrichment for inflammatory gene sets with the EMT also among the gene sets with an FDR<0.05 (table 4). As these data sets did not include values for plasma cytokines, we were not able to reproduce that analysis in these additional sets. Consequently, gene expression profiles from three tissues (muscle, lung and blood) in severe COPD show relatively greater inflammatory responses and increased EMT compared with mild COPD.

**Table 3:** Gene set enrichment for genes differentially expressed in the lung of patients with severe COPD compared with patients with mild COPD

Hallmark gene set	NES	FDR q-val
Positive enrichment		
Allograft rejection	2.43	<0.001
E2F targets	2.39	<0.001
MYC targets v2	2.27	0.001
IL6 JAK STA3 signalling	2.13	<0.001
Interferon gamma response	2.06	<0.001
G2M checkpoint	2.03	<0.001
Inflammatory response	2.01	<0.001
MTORC1 signalling	1.88	0.001
Complement	1.73	0.003
KRAS signalling up	1.70	0.005
Oxidative phosphorylation	1.69	0.005
Unfolded protein response	1.66	0.006
Glycolysis	1.64	0.008
Xenobiotic metabolism	1.59	0.011
EMT	1.59	0.012
Interferon alpha response	1.57	0.012
Coagulation	1.54	0.013
Reactive oxygen species pathway	1.47	0.025
Oestrogen response late	1.44	0.025
Spermatogenesis	1.41	0.029
IL2 STAT5 signalling	1.41	0.034
PI3K AKT mTOR signalling	1.39	0.034
MYC targets v1	1.34	0.045
Negative enrichment		
UV response down	−2.18	<0.001
TGF beta signalling	−2.05	<0.001
Myogenesis	−1.85	0.001
Hedgehog signalling	−1.78	0.003
Apical junction	−1.77	0.003
Wnt beta catenin signalling	−1.75	0.003
TNFa signalling via NFkb	−1.55	0.019
Oestrogen response early	−1.53	0.020
Apoptosis	−1.50	0.024
Protein secretion	−1.47	0.028
Notch signalling	−1.46	0.031

Differential gene expression was determined using LIMMA (severe–mild) and used to rank genes. The ranked genes were then analysed by GSEA using the Hallmark gene sets.

COPD, Chronic Obstructive Pulmonary disease; EMT, epithelial mesenchymal transition; FDR, false discovery rate; GSEA, Gene Set Enrichment Analysis; LIMMA, Linear Models for Microarray Data; NES, Normalised Enrichment Score.

**Table 4:** Gene set enrichment for genes differentially expressed in the blood of severe patients with COPD compared with patients with mild COPD

Hallmark gene set	NES	FDR q-val
Positive enrichment		
TNFA signalling via NFkb	2.16	<0.001
Inflammatory response	2.02	<0.001
IL6 JAK STAT3 signalling	1.92	0.001
Hypoxia	1.84	0.003
Cholesterol homeostasis	1.76	0.007
P53 pathway	1.74	0.007
Interferon alpha response	1.61	0.017
Wnt beta catenin signalling	1.61	0.016
Angiogenesis	1.60	0.016
Complement	1.59	0.016
Apical junction	1.58	0.017
Myogenesis	1.56	0.020
Coagulation	1.55	0.021
Oestrogen response early	1.53	0.022
EMT	1.53	0.021
Reactive oxygen species pathway	1.47	0.032
Notch signalling	1.46	0.033
Glycolysis	1.45	0.032
Xenobiotic metabolism	1.44	0.033
KRAS signalling up	1.43	0.035
Hedgehog signalling	1.41	0.040
Negative enrichment		
MYC targets v1	−2.89	<0.001
E2F targets	−2.38	<0.001
G2M checkpoint	−2.04	<0.001
MYC targets v2	−1.98	<0.001
Unfolded protein response	−1.81	<0.001
MTORC1 signalling	−1.51	0.015

Differential gene expression was determined using LIMMA (severe–mild) and used to rank genes. The ranked genes were then analysed by GSEA using the Hallmark gene sets.

COPD, Chronic Obstructive Pulmonary disease; EMT, epithelial mesenchymal transition; FDR, false discovery rate; GSEA, Gene Set Enrichment Analysis; LIMMA, Linear Models for Microarray Data; NES, Normalised Enrichment Score.

## Discussion

In this study, we analysed differences in gene expression profiles in patients with moderate and severe COPD to elucidate the associated loss of muscle mass in these patients. We initially showed that patients with more severe disease had a lower FFMI and FMI than patients with mild disease despite similar levels of physical activity. The lower FFMI and FMI in more severe disease is consistent with ECLIPSE, where patients with GOLD 4 COPD had a lower FFMI and BMI than those with GOLD 2 COPD.^29^

Gene expression profiles from quadriceps muscle biopsies in mild and severe COPD showed positive enrichment for inflammatory gene subsets (including TNF-α and IL6-STAT signalling), EMT and negative enrichment for gene sets associated with mitochondrial functions (fatty acid metabolism and oxidative phosphorylation). While the quadriceps of severe patients had a greater inflammatory load than those of mild patients, there were no differences in circulating levels of inflammatory cytokines between the two groups. The lack of difference in cytokines is not consistent with some studies which have reported an association of inflammatory cytokines with FEV_1_% predicted,^30 31^ in particular IL6. However, in the ECLIPSE study of over 1700 patients with COPD, there was no association with IL6 or TNF-α with baseline FEV_1_ and a positive association with IL8.^32^ Consistent with the variable findings from these data sets, the effect of actual levels of cytokines is likely to be small. Bradford *et al* estimate that IL-6 accounts for 4%–5% of the variability in FEV1% predicted; consequently, our study is underpowered to find such differences. However, our data add to the role of cytokines like IL-6 and IL-1b in COPD pathogenesis by suggesting that some individuals have a larger response to the same level of cytokine than others. This variability in response will reduce the observed association of any individual cytokine with disease progression. In our data set, the inclusion of controls produced negative associations of cytokines with FEV_1_ % predicted indicative of systemic inflammation (online supplemental table S3), but these were not present when the controls were excluded.

In severe patients, both proinflammatory and anti-inflammatory cytokines were positively associated with inflammatory response gene sets, whereas in mild patients, anti-inflammatory cytokines were negatively associated with the same gene sets. These data imply either that patients with severe COPD are more sensitive to proinflammatory cytokines than those with mild COPD or that they are less sensitive to anti-inflammatory cytokines. Comparison of gene expression profiles in blood and lung samples implied that this difference in inflammatory sensitivity was not limited to skeletal muscle. This greater responsiveness of patients with severe COPD to inflammatory stimuli appears to lead to more rapid loss of muscle.

Consistent with greater sensitivity to proinflammatory signals, patients with more severe disease had higher expression of inflammatory cytokine receptors and signal transducers, and expression of IL31RA and STAM not only contributed to inflammatory gene sets associated with lung function, but also associated negatively with FFMI.

In muscle and lung tissue, in addition to the inflammatory gene and EMT gene sets, gene sets associated with the transcription factor MYC were also enriched in those with more severe disease. MYC promotes both the EMT and inflammation, and in muscle, we found that MYC expression was proportional to MCP-1, IL6, IL31RA and OSMR expression. In the blood, the associations with MYC gene sets were in the opposite direction with higher expression in those with mild COPD. Our analysis showing increased inflammatory gene expression from IL6/STAT/JAK and TNF-α signalling pathways and reduced expression of MYC gene sets in the blood of patients with severe COPD is consistent with a study developing a blood transcriptional risk score for decline in lung function and susceptibility to COPD.^33^ The mechanisms causing differences in enrichment for the MYC gene sets in blood and muscle/lung are unclear, but it seems likely that they reflect differences in the role of MYC in the tissues in response to disease.

The increased expression of EMT genes is likely to reflect a fibrotic response that is part of an overall ‘repair’ response of the tissue to injury. Our previous study showed that patients with COPD have a significant increase in genes associated with the extracellular matrix and repair, including periostin (POSTN), a gene that contributes to the EMT gene set we identify here.^21^ The coagulation gene set was also enriched. This data set may also reflect a role in repair/fibrosis, as has been suggested in fibrosis in chronic inflammatory conditions.^34 35^

We cannot determine whether patients become more sensitive to inflammation as disease progresses or whether those that are intrinsically more sensitive progress through the disease more rapidly. However, several lines of evidence point towards the latter. First, patients with severe COPD must have had mild disease at some point, suggesting that they should be older than those with mild COPD. In our cohort, the reverse was true despite similar levels of smoking. Furthermore, the rate of change in FEV_1_ in patients with COPD has been shown to be very variable.^32^ In the cohort analysed for lung cell gene expression, neither age nor smoking status was reported, and in the cohort analysed for blood cell gene expression, age did not differ between those with mild and those with severe COPD, but there were no data relating to smoking history. In the ECLIPSE study, there was no difference in the ages of individuals with more severe COPD than those with mild COPD.^29^ Second, the observation is consistent across three tissue types, suggesting that this feature is not unique to muscle. Finally, polymorphisms in inflammatory genes have been associated with the development of COPD. Indeed, ingenuity pathway analysis of data from the GWAS meta-analysis identified not only inflammatory gene sets (eg, STAT3 signalling, IL8 signalling, IL15 production) but also those associated with EMT, wound healing and MYC mediated apoptosis signalling.^12^

Our data have most relevance to muscle loss and suggest that inflammation is a key component of muscle loss in COPD. It is interesting that despite markedly lower activity levels, patients with mild COPD had similar FFMI to controls. Indeed, the tendency of these patients to have a higher FMI suggests that inactivity contributes to adiposity. In patients with severe COPD, both FFMI and FMI are reduced compared with patients with mild COPD despite similar levels of activity, indicating that these patients are sarcopenic.

The greatest association with inflammatory response gene sets in the muscle was shown by IL1β, suggesting that it is a major driver of the inflammatory response in severe COPD. However, the increase in IL1ß compared with controls is small, making it possible that IL1ß is just correlating with other more important cytokines. Alternatively, it is possible that the response observed in people with severe disease is the combined response to all the proinflammatory cytokines present. Consistent with this observation, several studies have also linked individual cytokines or combinations with muscle mass in studies of patients with chronic disease, whereas other studies have failed to find such an association.^36^,^39^

The strongest association of disease severity with receptor expression was with IL31RA and its coreceptor OSMR, raising the possibility that IL31 and/or oncostatin M (OSM) are important regulators of muscle wasting. Unfortunately, we were unable to quantify IL31 or OSM in the blood of these patients as we no longer have the appropriate samples. IL31RA is expressed in a restricted range of tissues including peripheral blood lymphocytes and skeletal muscle (https://www.proteinatlas.org/ENSG00000164509-IL31RA/tissue). It is, therefore, possible that IL31 promotes muscle atrophy either directly or through the recruitment of lymphocytes that subsequently promote atrophy through other pathways. However, to our knowledge, the contribution of IL31 to muscle wasting has yet to be tested. By contrast, OSM promoted muscle atrophy in vitro and in murine models^40^ and may contribute to muscle wasting in cancer.^41^ Furthermore, increased OSM was detected in patients with COPD and in a recent study of inflammatory markers in patients with COPD, OSM-associated pathways were implicated in the pathology of COPD, although OSM itself was not quantified.^42^ The role of these proteins in COPD-associated muscle wasting, therefore, remains to be determined.

As indicated above, the patients with more severe disease had lower expression of genes associated with mitochondrial function (oxidative phosphorylation and fatty acid metabolism). Furthermore, a negative association of these mitochondrial genes with several of the cytokines was present within the severe disease group (eg, with IL2, IL8, interferon-γ gamma and TNF-α, online supplemental tables S5 and S9–S11). Mitochondrial dysfunction is associated with muscle loss in a range of conditions including COPD.^43 44^ The associated reduction in energy provision may well contribute to wasting and will also contribute to the reduced exercise capacity of the patients. Our previous studies have shown that quadriceps expression of miRNAs that promote mitochondrial ribosome stress leading to mitochondrial dysfunction is inversely proportional to lung function in these patients providing a possible mechanism.^45 46^ Whether these miRNAs are regulated by inflammatory cytokines remains to be established, but the data provided in this paper suggest that identifying individual associations of any individual cytokine would be complicated by the sensitivity of individuals to the stimulus.

Several anti-inflammatory agents have been trialled in COPD with limited success.^47 48^ Targets included IL-1β, which we show here is the agent with the strongest association with muscle inflammatory gene signalling. Negative results of these studies may indicate the limited importance of inflammation in the progression of COPD, contrary to our suggestion. However, there are several other potential reasons for the apparent failure of this approach. The first is kinetics: the trials may simply not have been long enough to reach their primary end points. The second is whether inhibiting inflammation can reverse damage already done, as improvement in lung function is one end point that has been tested. Stabilising function may be the best that can be achieved with this approach. Third, in terms of muscle mass, the studies done to date have focused on lung function rather than muscle mass, making it possible that antagonism of IL-1 signalling may improve muscle mass but not affect lung function. Finally, the data from this paper raise the possibility that only a subset of patients would respond significantly to anti-IL1 therapy so that preselection of responsive patients might show beneficial effects of such therapy.

### Limitations of the study

This study is cross-sectional so does not demonstrate causative effects of the changes in gene expression and the association with FFMI. To further understand this process, analysis of the muscle inflammatory profile in younger individuals with early-stage COPD followed by a longitudinal assessment of their disease progression and muscle atrophy would provide the next step. It would then be possible to determine the effect of inhibiting inflammation in those with the greatest sensitivity to inflammation. Furthermore, FFMI is a surrogate for muscle mass, and so the data should be interpreted with caution. However, for a study of muscle gene expression in disease, the cohorts that we have examined are comparatively large. We have focused on gene expression in patients alone, comparing mild and severe COPD, to reduce the effects of activity that occur if controls are used as the comparator. This analysis, therefore, limits the generalisability of our findings. We did not examine the associations of gene expression with physical performance in this study. Our analysis of the data from lung and blood samples is also limited by the availability of data from those studies. We cannot determine whether there were differences in cytokine levels between mild and severe patients in these groups, nor compare cytokine levels with gene expression profiles, as we were able to do in the skeletal muscle study. However, the overall expression profiles concur with our general findings of greater sensitivity of patients with severe disease to inflammatory signalling. This interpretation is further supported by the observation that TNF-α production by blood cells in response to bacterial lipopolysaccharide was greater in patients with severe compared with mild COPD.^49^

Our demonstration of associations of circulating cytokines with the levels of target transcription pathways in the muscle of patients with severe disease is suggestive of a direct effect of these cytokines on muscle phenotype. That we see similar transcription profiles in other tissues is consistent with this suggestion, as are studies of the effects of exogenous cytokines on muscle loss in animals. These data also support the idea that there is communication between the diseased lung and the muscle to promote the use of muscle as a resource for repair and the immune response,^50^ or that there is inflammation spill-over from the lung to muscle contributing to muscle loss, though our studies suggest that this is modified by factors that control the sensitivity of individuals to such stimuli. However, we did not measure tissue cytokines which are also likely to contribute to the overall loss of muscle.

The advantage of studies based on transcriptomics is the breadth of analytes that can be measured so that an overall picture can be generated and the consequent identification of potential targets and pathways. However, a significant limitation is that RNA levels do not always reflect the levels of proteins in the tissue. The relative contribution of tissue-derived inflammatory cytokines is, therefore, difficult to determine. Unfortunately, we no longer have the samples available to identify differences in protein levels from these patients so further studies will be required to determine whether the levels of key proteins in the identified pathways differ.

## Conclusions

Our study shows that patients with COPD with more advanced disease have greater inflammatory gene expression in their quadriceps muscles despite similar levels of circulating inflammatory cytokines. This appears to be due to lower sensitivity to anti-inflammatory cytokines and/or higher sensitivity to proinflammatory cytokines in patients with more severe disease. Our data suggest that this occurs because of higher expression of receptors and signalling components of the proinflammatory pathways. These differences may be intrinsic to the individual rather than having developed during the disease process because the severe COPD cohort was not older than the mild group. This further suggests that targeting inflammation in selected patients may be an appropriate strategy to prevent muscle atrophy and progressive COPD. Confirmation of this observation will require analysis in a longitudinal cohort of patients.

## Supplementary material

10.1136/thorax-2024-221901 online supplemental file 1

10.1136/thorax-2024-221901 online supplemental file 2

10.1136/thorax-2024-221901 online supplemental file 3

10.1136/thorax-2024-221901 online supplemental file 4

## Data Availability

Data are available on reasonable request.
