# Supplementary material for: Variability in sensitivity to inflammation in muscle and lung of patients with COPD may underlie susceptibility to lung function decline
Source: Thorax. 2025 Apr 16;80(8):e221901. doi: 10.1136/thorax-2024-221901 (PMC12322413; doi:10.1136/thorax-2024-221901)
Supplement: online supplemental file 1 [file thorax-80-8-s001.pdf]

**Supplementary methods:**

**Gene set enrichment (preranked).** Gene set enrichment analysis takes a ranked list of genes and determines whether the presence of genes from predetermined sets (e.g. genes associated with inflammatory signalling) at either end of the list is statistically different from the expected distribution of those genes. The software calculates the net enrichment score (NES) of each of the defined set of genes at the leading or trailing edge of the list. Statistical significance is determined by comparing the NES for the gene set with that generated by randomising the list provided 1000 times to generate a null set. The FDR is the probability that the NES is a false positive. Our gene ranks were produced by ranking the genes based on differential expression between mild and severe COPD when comparing groups or by using the biweight midcorrelation coefficient as the ranking metric.

**Supplementary results:**

As men and women have markedly different FFMI and FMI, we repeated all analyses in the male patients alone. This analysis gave the same results for FFMI and activity with one additional observation; in the patients with mild disease FMI was higher than either of the other two groups (Fig. S1 and Table S12). These data suggest that in patients with mild COPD the reduction in activity leads to an increase in adiposity rather than a loss of muscle mass.

Analysis of the inflammatory cytokines between the two patient groups and controls in the male patients also showed that both groups had increased levels of CRP, TNFα, IL1β, IL2, IL4, IL5, IL6, IL8 and IL10 than controls but the levels between the two disease groups did not differ (Table S13).

**Bioinformatic Analysis of male patients alone**

Sex has a significant effect on fibre proportions and will therefore have an effect on gene expression profile. Sex differences also affect inflammatory cytokine levels and inflammatory processes. Therefore, to determine whether sex differences contributed to our findings, we also repeated our bio-informatic analysis comparing the transcriptome in males only with $TLCO_{\%pred}$, and for males with mild or severe COPD with IL1β and IL10 as the cytokines showing the strongest associations.

In accordance with the observations from the whole cohort, comparison of the genes most tightly associated with $TLCO_{\%pred}$ showed that those most positively associated were enriched for genes from EMT and inflammatory gene-sets, and those most negatively associated were from the gene-sets associated with oxidative phosphorylation and mitochondrial function (Table S14). In the males with severe COPD, IL1β was positively associated with genes from EMT and inflammatory gene sets as observed in the full cohort (Table S15). In the males with mild COPD, there was no positive enrichment for any gene sets with IL1β and negative enrichment for genes associated with oxidative phosphorylation (Table S18). Analysis of IL10 showed no positive enrichment for with inflammatory gene-sets in those with severe COPD, but a strong negative enrichment for these gene sets in male patients with mild COPD (Table S16).

Consequently, this analysis is consistent with the suggestion that in patients with severe COPD the response to pro-inflammatory cytokines predominates, whereas in those with mild disease the anti-inflammatory response predominates. Hence there was no obvious sex difference on our analysis. We did not repeat this analysis in the samples from females because of the relatively small numbers available.
