# Supplementary material for: Variability in sensitivity to inflammation in muscle and lung of patients with COPD may underlie susceptibility to lung function decline
Source: Thorax. 2025 Apr 16;80(8):e221901. doi: 10.1136/thorax-2024-221901 (PMC12322413; doi:10.1136/thorax-2024-221901)
Supplement: online supplemental file 2 [file thorax-80-8-s002.pdf]

**Supplementary tables**

**Table S1 Demographics for the entire cohort subdivided by disease severity**

| | **Control** | **Mild** | **Severe** | **P value** |
|---|---|---|---|---|
| **m/f** | 16/14 | 17/11 | 35/17 | |
| **Age (yrs)** | 67 (65, 72) | 74 (64, 78) †† | 64 (59, 69) | 0.003 |
| **Height (cm)** | 167 (164, 173) | 167 (158, 173) | 167 (163, 173) | 0.709 |
| **BMI (kg/m²)** | 24.8 (24.0, 28.0) | 26.8 (23.6,30.1) †† | 23.7 (21.3, 26.2) | 0.004 |
| **FFMI (kg/m²)** | 16.5 (15.3, 20) | 17.1 (15.6, 18.5)†† | 15.4 (14.5, 16.8)* | 0.002 |
| **FMI (kg/m²)** | 8.6 (6.8, 10.4) | 9.6 (8.1, 12.2) | 8.0 (6.7, 9.9) | 0.07 |
| **Pack-year history** | 3 (0, 15) | 45 (40, 64) *** | 45 (30, 63) *** | <0.001 |
| **$FEV_1$ (% pred)** | 108.5 (100.4, 113.3) | 62.5(56.5, 71.3) **,††† | 33 (26, 43) *** | <0.001 |
| **$TL_{CO}$ (% pred)** | 87.1 (80.2, 95.3) | 55.6 (47.6, 63.8)***,†† | 33 (26, 47)*** | <0.001 |
| **Locomotion time (mins)** | 95 (61, 138) | 45 (38, 63)** | 37 (23, 61)*** | <0.001 |
| **Movement intensity (m/s)** | 2.28 (1.78, 2.79) | 1.72 (1.44, 2.05)** | 1.66 (1.43, 1.90)** | 0.001 |

P value = Significance across the groups was calculated Kruskal-Wallis test. Differences between group pairs were calculated using Dunn-Bonferroni test for multiple comparisons. For mild or severe vs control *p<0.05, **, p<0.01, ***<0.001, for mild vs severe †p<0.05, †† p<0.01, †††p<0.001. Data are shown as median (inter quartile range).

**Table S2 Demographics for the Array cohort subdivided by disease severity**

| | **Mild** | **Severe** | **P value** |
|---|---|---|---|
| **m/f** | 17/11 | 34/17 | |
| **Age (yrs)** | 74 (64, 78) | 64 (59, 69) | <0.001 |
| **Height (cm)** | 167 (158, 173) | 167 (163, 173) | 0.512 |
| **BMI (kg/m2)** | 26.8 (23.6, 30.1) | 23.7 (21.3, 26.2) | 0.002 |
| **FFMI (kg/m2)** | 17.1 (15.6, 18.5) | 15.4 (14.5, 16.8) | 0.003 |
| **FMI (kg/m2)** | 9.6 (8.1, 12.2) | 8.0 (6.7, 9.9) | 0.022 |
| **Pack-year history** | 45 (40, 64) | 45 (30, 62.5) | 0.620 |
| **FEV1 (% pred)** | 62.5 (56.5, 71.3) | 33.4 (26.1, 47.3) | <0.001 |
| **TLCO (% pred)** | 55.6 (47.6, 63.8) | 32.8 (25.5, 47.3) | <0.001 |
| **Locomotion time (mins)** | 45 (38, 63) | 37 (23, 61) | 0.109 |
| **Movement intensity (m/s)** | 1.72 (1.44, 2.05) | 1.66 (*1.43, 1.90) | 0.858 |

P value = Significance across the groups was calculated by Mann Whitney U test. Data are shown as median (inter quartile range)

**Table S3: Gene set enrichment for gene sets that associate with lung function measured as $TL_{CO}$ % predicted.**

| ALL COPD | | |
|---|---|---|
| TLCO% pred | | |
| **Hallmark gene set** | **NES** | **FDR q-val** |
| **Negative enrichment** | | |
| EPITHELIAL MESENCHYMAL TRANSITION | -2.28 | <0.001 |
| IL6 JAK STAT3 SIGNALLING | -1.99 | <0.001 |
| INTERFERON GAMMA RESPONSE | -1.93 | <0.001 |
| DNA REPAIR | -1.79 | 0.003 |
| E2F TARGETS | -1.76 | 0.005 |
| UV RESPONSE DN | -1.76 | 0.004 |
| INTERFERON ALPHA RESPONSE | -1.74 | 0.005 |
| MYC TARGETS V2 | -1.72 | 0.005 |
| APICAL JUNCTION | -1.72 | 0.005 |
| IL2 STAT5 SIGNALLING | -1.71 | 0.005 |
| PROTEIN SECRETION | -1.60 | 0.012 |
| ANGIOGENESIS | -1.59 | 0.011 |
| P53 PATHWAY | -1.53 | 0.019 |
| UNFOLDED PROTEIN RESPONSE | -1.49 | 0.025 |
| ALLOGRAFT REJECTION | -1.47 | 0.031 |
| **Positive enrichment** | | |
| OXIDATIVE PHOSPHORYLATION | 3.19 | <0.001 |
| FATTY ACID METABOLISM | 2.64 | <0.001 |
| ADIPOGENESIS | 2.35 | <0.001 |
| REACTIVE OXYGEN SPECIES PATHWAY | 1.94 | 0.001 |
| PEROXISOME | 1.74 | 0.003 |
| BILE ACID METABOLISM | 1.56 | 0.016 |

**NES normalised enrichment score**

Note in this table enrichment is with lung function and therefore is the opposite way round to the differential gene expression analysis between severe and mild patients

**Table S4A: Circulating levels of the inflammatory markers in controls and the patient groups**

| | Control | Mild | Severe | P value |
|---|---|---|---|---|
| CRP (mg/mL) | 0.18 (0.11, 0.26) | 0.94 (0.48, 2.36)*** | 0.55 (0.25, 1.24)*** | <0.001 |
| TNF-α (pg/mL) | 13.4 (1.3, 32.1) | 30.3 (22.9, 45.1) * | 42.7 (30.0, 58.3) ** | <0.001 |
| IL1β (pg/mL) | 0.21 (0.17, 0.31) | 0.44 (0.34, 0.58) ** | 0.48 (0.39, 0.62) *** | <0.001 |
| IL2 (pg/mL) | 33 (7, 52.6) | 89 (66, 120) *** | 108 (78, 144) *** | <0.001 |
| IL4 (pg/mL) | 20.2 (5.2, 30.9) | 30.9 (21.7, 45.1) * | 40.7 (28.6, 58.4) *** | <0.001 |
| IL5 (pg/mL) | 2.1 (1.1, 4.1) | 8.4 (4.4, 13.7) *** | 8.9 (5.7, 12.8) *** | <0.001 |
| IL6 (pg/mL) | 3.8 (1.3, 7.1) | 7.6 (4.6, 13.7) ** | 9.3 (6.0, 13.6) *** | <0.001 |
| IL8 (pg/mL) | 8.5 (6.3, 13.8) | 19.5 (16.6, 27.8) *** | 25.3 (16.6, 33.3) *** | <0.001 |
| IL10 (pg/mL) | 2.3 (1.3, 5.4) | 6.9 (4.4, 10.1) ** | 10.1 (5.6, 14.5) *** | <0.001 |
| INF-γ (pg/mL) | 23 3 (9.48, 38.7) | 33.7 (21.8, 45.4) * | 39.6 (26.4, 53.9) ** | <0.001 |

P value = Significance across the groups was calculated by Kruskal-Wallis test. Differences between group pairs were calculated using Dunn-Bonferroni test for multiple comparisons. For mild or severe vs control *p0.05, **, p<0.01, ***<0.001. **No differences were observed between mild and severe disease groups**. Data are shown as median (inter quartile range) as the data did not show a normal distribution.

**Table S4B: Correlation coefficients for CRP and cytokines with FEV1%**

| | vs FEV1% with controls | | vs FEV1% patients only | |
|---|---|---|---|---|
| | rho | P | rho | P |
| CRP (mg/mL) | -0.270 | 0.001 | 0.117 | 0.234 |
| TNF-$\alpha$ (pg/mL) | -0.153 | 0.068 | 0.086 | 0.357 |
| IL1$\beta$ (pg/mL) | -0.277 | 0.002 | -0.058 | 0.546 |
| IL2 (pg/mL) | -0.224 | 0.009 | 0.099 | 0.298 |
| IL4 (pg/mL) | -0.186 | 0.027 | 0.033 | 0.728 |
| IL5 (pg/mL) | -0.179 | 0.032 | 0.162 | 0.086 |
| IL6 (pg/mL) | -0.221 | 0.009 | 0.047 | 0.614 |
| IL8 (pg/mL) | -0.155 | 0.063 | 0.162 | 0.084 |
| IL10 (pg/mL) | -0.246 | 0.004 | 0.029 | 0.756 |
| INF-$\gamma$ (pg/mL) | -0.097 | 0.267 | 0.066 | 0.496 |

Circulating levels of inflammatory cytokines were compared with lung function using Spearman's correlation. Correlation coefficients (rho) and associated p values are given.

**Table S5: Gene set enrichment for Genes correlating with circulating IL2 in patients at different disease stages**

| MILD COPD | | | Severe COPD | | |
|---|---|---|---|---|---|
| **IL2** | | | **IL2** | | |
| Hallmark Gene Set | NES | FDR q-val | Hallmark Gene Set | NES | FDR q-val |
| **Positive enrichment** | | | **Positive enrichment** | | |
| NOTHING | | | ALLOGRAFT REJECTION | 2.28 | <0.001 |
| | | | TNFA SIGNALING VIA NFKB | 1.98 | 0.001 |
| | | | IL6 JAK STAT3 SIGNALING | 1.72 | 0.009 |
| | | | INFLAMMATORY RESPONSE | 1.68 | 0.012 |
| | | | INTERFERON GAMMA RESPONSE | 1.58 | 0.021 |
| **Negative enrichment** | | | **Negative enrichment** | | |
| ALLOGRAFT REJECTION | -1.73 | 0.026 | OXIDATIVE PHOSPHORYLATION | -1.99 | 0.002 |
| IL6 JAK STAT3 SIGNALING | -1.65 | 0.032 | FATTY ACID METABOLISM | -1.98 | 0.001 |
| INFLAMMATORY RESPONSE | -1.59 | 0.036 | ADIPOGENESIS | -1.94 | 0.001 |
| INTERFERON ALPHA RESPONSE | -1.55 | 0.039 | MYC TARGETS V2 | -1.80 | 0.003 |
| INTERFERON GAMMA RESPONSE | -1.52 | 0.044 | BILE ACID METABOLISM | -1.74 | 0.005 |
| | | | APICAL SURFACE | -1.57 | 0.028 |
| | | | | | |
| | | | | | |

**Table S6: Gene set enrichment for Genes correlating with circulating IL4 in patients at different disease stages**

| MILD COPD | | | Severe COPD | | |
|---|---|---|---|---|---|
| **IL4** | | | **IL4** | | |
| Hallmark Gene Set | NES | FDR q-val | Hallmark Gene Set | NES | FDR q-val |
| **Positive enrichment** | | | **Positive enrichment** | | |
| EPITHELIAL MESENCHYMAL TRANSITION | 2.01 | 0.001 | TNFA SIGNALING VIA NFKB | 2.21 | <0.001 |
| OXIDATIVE PHOSPHORYLATION | 1.87 | 0.002 | ALLOGRAFT REJECTION | 2.20 | 0.000 |
| PANCREAS BETA CELLS | 1.71 | 0.008 | HEME METABOLISM | 1.80 | 0.008 |
| CHOLESTEROL HOMEOSTASIS | 1.69 | 0.007 | INFLAMMATORY RESPONSE | 1.65 | 0.024 |
| GLYCOLYSIS | 1.63 | 0.009 | | | |
| APICAL JUNCTION | 1.55 | 0.021 | **Negative enrichment** | | |
| ESTROGEN RESPONSE LATE | 1.55 | 0.027 | MYC TARGETS V1 | -1.93 | 0.004 |
| COAGULATION | 1.49 | 0.032 | MYC TARGETS V2 | -1.92 | 0.002 |
| FATTY ACID METABOLISM | 1.48 | 0.034 | PROTEIN SECRETION | -1.70 | 0.013 |
| | | | E2F TARGETS | -1.66 | 0.014 |
| | | | INTERFERON ALPHA RESPONSE | -1.57 | 0.029 |
| **Negative enrichment** | | | UNFOLDED PROTEIN RESPONSE | -1.54 | 0.033 |
| ALLOGRAFT REJECTION | -2.24 | <0.001 | G2M CHECKPOINT | -1.51 | 0.038 |
| HEME METABOLISM | -1.91 | 0.005 | PEROXISOME | -1.49 | 0.039 |
| PROTEIN SECRETION | -1.79 | 0.005 | DNA REPAIR | -1.49 | 0.036 |
| INFLAMMATORY RESPONSE | -1.55 | 0.032 | MITOTIC SPINDLE | -1.46 | 0.038 |

**Table S7: Gene set enrichment for Genes correlating with circulating IL5 in patients at different disease stages**

| MILD COPD | | | Severe COPD | | |
|---|---|---|---|---|---|
| **IL5** | | | **IL5** | | |
| Hallmark Gene Set | NES | FDR q-val | Hallmark Gene Set | NES | FDR q-val |
| **Positive enrichment** | | | **Positive enrichment** | | |
| NOTHING | | | ALLOGRAFT REJECTION | 1.97 | 0.001 |
| | | | TNFA SIGNALING VIA NFKB | 1.61 | 0.033 |
| **Negative enrichment** | | | **Negative enrichment** | | |
| ALLOGRAFT REJECTION | -2.08 | <0.001 | ADIPOGENESIS | -1.89 | 0.004 |
| IL6 JAK STAT3 SIGNALING | -2.02 | <0.001 | FATTY ACID METABOLISM | -1.87 | 0.003 |
| INTERFERON ALPHA RESPONSE | -1.73 | 0.007 | | | |
| KRAS SIGNALING UP | -1.72 | 0.008 | | | |
| INTERFERON GAMMA RESPONSE | -1.71 | 0.007 | | | |
| INFLAMMATORY RESPONSE | -1.66 | 0.010 | | | |
| HEME METABOLISM | -1.56 | 0.026 | | | |
| APICAL SURFACE | -1.53 | 0.031 | | | |
| COMPLEMENT | -1.53 | 0.029 | | | |
| WNT BETA CATENIN SIGNALING | -1.51 | 0.033 | | | |

**Table S8: Gene set enrichment for Genes correlating with circulating IL6 in patients at different disease stages**

| MILD COPD | | | Severe COPD | | |
|---|---|---|---|---|---|
| **IL6** | | | **IL6** | | |
| Hallmark Gene Set | NES | FDR q-val | Hallmark Gene Set | NES | FDR q-val |
| **Positive enrichment** | | | **Positive enrichment** | | |
| KRAS SIGNALING DN | 1.72 | 0.029 | EPITHELIAL MESENCHYMAL TRANSITION | 2.47 | <0.001 |
| | | | MTORC1 SIGNALING | 2.11 | <0.001 |
| | | | COAGULATION | 2.06 | 0.001 |
| | | | APOPTOSIS | 1.98 | 0.001 |
| | | | HEME METABOLISM | 1.90 | 0.002 |
| | | | ANGIOGENESIS | 1.89 | 0.001 |
| | | | TNFA SIGNALING VIA NFKB | 1.85 | 0.002 |
| | | | IL6 JAK STAT3 SIGNALING | 1.77 | 0.005 |
| | | | ALLOGRAFT REJECTION | 1.71 | 0.007 |
| | | | ANDROGEN RESPONSE | 1.67 | 0.011 |
| | | | PANCREAS BETA CELLS | 1.59 | 0.019 |
| | | | P53 PATHWAY | 1.55 | 0.026 |
| | | | PROTEIN SECRETION | 1.49 | 0.036 |
| | | | UV RESPONSE UP | 1.46 | 0.045 |
| | | | COMPLEMENT | 1.44 | 0.048 |
| **Negative enrichment** | | | **Negative enrichment** | | |
| PROTEIN SECRETION | -2.20 | <0.001 | BILE ACID METABOLISM | -1.67 | 0.018 |
| MITOTIC SPINDLE | -2.20 | <0.001 | WNT BETA CATENIN SIGNALING | -1.51 | 0.043 |
| E2F TARGETS | -2.14 | <0.001 | KRAS SIGNALING DN | -1.46 | 0.047 |
| G2M CHECKPOINT | -2.14 | <0.001 | | | |
| MYC TARGETS V1 | -1.80 | 0.003 | | | |
| WNT BETA CATENIN SIGNALING | -1.59 | 0.016 | | | |
| DNA REPAIR | -1.52 | 0.028 | | | |
| HEME METABOLISM | -1.50 | 0.029 | | | |
| UNFOLDED PROTEIN RESPONSE | -1.47 | 0.035 | | | |
| UV RESPONSE DN | -1.46 | 0.034 | | | |
| TGF BETA SIGNALING | -1.44 | 0.036 | | | |
| | | | | | |

**Table S9: Gene set enrichment for Genes correlating with circulating IL8 in patients at different disease stages**

| MILD COPD | | | Severe COPD | | |
|---|---|---|---|---|---|
| **IL8** | | | **IL8** | | |
| Hallmark Gene Set | NES | FDR q-val | Hallmark Gene Set | NES | FDR q-val |
| **Positive enrichment** | | | **Positive enrichment** | | |
| EPITHELIAL MESENCHYMAL TRANSITION | 2.54 | <0.001 | ALLOGRAFT REJECTION | 2.33 | <0.001 |
| CHOLESTEROL HOMEOSTASIS | 1.98 | <0.001 | TNFA SIGNALING VIA NFKB | 1.95 | <0.001 |
| ANGIOGENESIS | 1.97 | <0.001 | IL6 JAK STAT3 SIGNALING | 1.80 | <0.001 |
| NOTCH SIGNALING | 1.78 | 0.005 | INFLAMMATORY RESPONSE | 1.60 | 0.020 |
| APICAL JUNCTION | 1.75 | 0.006 | HEME METABOLISM | 1.59 | 0.019 |
| HYPOXIA | 1.69 | 0.009 | INTERFERON GAMMA RESPONSE | 1.47 | 0.046 |
| COAGULATION | 1.67 | 0.010 | APOPTOSIS | 1.45 | 0.045 |
| GLYCOLYSIS | 1.60 | 0.015 | ESTROGEN RESPONSE LATE | 1.44 | 0.044 |
| MTORC1 SIGNALING | 1.48 | 0.040 | | | |
| TNFA SIGNALING VIA NFKB | 1.48 | 0.036 | | | |
| **Negative enrichment** | | | **Negative enrichment** | | |
| E2F TARGETS | -1.65 | 0.043 | MYC TARGETS V2 | -1.94 | 0.003 |
| HEME METABOLISM | -1.57 | 0.041 | OXIDATIVE PHOSPHORYLATION | -1.91 | 0.002 |
| | | | FATTY ACID METABOLISM | -1.77 | 0.007 |
| | | | ADIPOGENESIS | -1.70 | 0.011 |
| | | | E2F TARGETS | -1.64 | 0.015 |
| | | | BILE ACID METABOLISM | -1.60 | 0.018 |
| | | | MYC TARGETS V1 | -1.51 | 0.041 |
| | | | UNFOLDED PROTEIN RESPONSE | -1.48 | 0.047 |
| | | | G2M CHECKPOINT | -1.48 | 0.042 |
| | | | | | |

**Table S10: Gene set enrichment for Genes correlating with circulating INFg in patients at different disease stages**

| MILD COPD | | | Severe COPD | | |
|---|---|---|---|---|---|
| **INFg** | | | **INFg** | | |
| Hallmark Gene Set | NES | FDR q-val | Hallmark Gene Set | NES | FDR q-val |
| **Positive enrichment** | | | **Positive enrichment** | | |
| CHOLESTEROL HOMEOSTASIS | 1.81 | 0.013 | ALLOGRAFT REJECTION | 2.23 | <0.001 |
| TNFA SIGNALING VIA NFKB | 1.76 | 0.017 | TNFA SIGNALING VIA NFKB | 1.87 | 0.006 |
| HYPOXIA | 1.68 | 0.020 | INFLAMMATORY RESPONSE | 1.59 | 0.028 |
| | | | HEME METABOLISM | 1.52 | 0.043 |
| **Negative enrichment** | | | **Negative enrichment** | | |
| ALLOGRAFT REJECTION | -1.69 | 0.032 | FATTY ACID METABOLISM | -1.93 | 0.002 |
| | | | BILE ACID METABOLISM | -1.93 | 0.001 |
| | | | ADIPOGENESIS | -1.83 | 0.003 |
| | | | OXIDATIVE PHOSPHORYLATION | -1.81 | 0.003 |
| | | | | | |

**Table S11: Gene set enrichment for Genes correlating with circulating TNFa in patients at different disease stages**

| MILD COPD | | | Severe COPD | | |
|---|---|---|---|---|---|
| **TNFa** | | | **TNFa** | | |
| Hallmark Gene Set | NES | FDR q-val | Hallmark Gene Set | NES | FDR q-val |
| **Positive enrichment** | | | **Positive enrichment** | | |
| TNFA SIGNALING VIA NFKB | 1.59 | 0.046 | ALLOGRAFT REJECTION | 2.10 | <0.001 |
| ANGIOGENESIS | 1.58 | 0.037 | TNFA SIGNALING VIA NFKB | 1.87 | 0.002 |
| EPITHELIAL MESENCHYMAL TRANSITION | 1.54 | 0.044 | | | |
| | | | | | |
| | | | | | |
| **Negative enrichment** | | | **Negative enrichment** | | |
| ALLOGRAFT REJECTION | -2.04 | 0.001 | BILE ACID METABOLISM | -1.83 | 0.010 |
| | | | FATTY ACID METABOLISM | -1.67 | 0.025 |
| | | | ADIPOGENESIS | -1.65 | 0.018 |
| | | | INTERFERON ALPHA RESPONSE | -1.60 | 0.024 |
| | | | | | |
| | | | | | |

**Table S12 Male patients only.**

| | Mild | Severe | P value |
|---|---|---|---|
| Age (yrs) | 74 (64, 78) | *64 (59, 69)* | 0.005 |
| Height (cm) | 173 (170, 176) | *173 (168, 177)* | 0.734 |
| BMI (kg/m$^2$) | 26.8 (24.7,30.9) | *23.3 (21.3, 25.4)* | 0.003 |
| FFMI (kg/m$^2$) | 17.6 (16.5, 19.7) | *15.5 (14.6, 17.0)* | 0.001 |
| FMI (kg/m$^2$) | 9.4 (8.0, 10.8) | 7.7 (6.0, 9.1) | 0.028 |
| Pack-year history | 52 (41, 82) | 46 (30, 69) | 0.490 |
| $FEV_1$ (% pred) | 62.3(57.2, 75.8) | 31.6 (24.5, 42.5) | <0.001 |
| $TL_{CO}$ (% pred) | 60.1 (44.9, 68.8) | 32.3 (25.6, 53.3) | <0.001 |
| Locomotion time (mins) | 46 (38, 87) | 39 (18, 68) | 0.106 |
| Movement intensity | 1.91 (1.54, 2.23)** | 1.75 (1.51, 1.93)** | 0.203 |

**Table S13 Inflammation male patients only**

| | Mild | Severe | P value |
|---|---|---|---|
| CRP (mg/mL) | 0.68 (0.38, 1.99) | 0.38 (0.16, 0.99) | 0.193 |
| TNF-α (pg/mL) | 30.3 (22.9, 45.1) | 42.7 (30.0, 58.3) | 0.201 |
| IL1β (pg/mL) | 0.39 (0.33, 0.58) | 0.50 (0.40, 0.67) | 0.163 |
| IL2 (pg/mL) | 100 (68, 143) | 110 (76, 141) | 0.780 |
| IL4 (pg/mL) | 31.3 (20.1, 46.6) | 41.3 (28.2, 56.5) | 0.127 |
| IL5 (pg/mL) | 8.4 (4.3, 13.8) | 10.0 (5.4, 14.0) | 0.780 |
| IL6 (pg/mL) | 8.1 (4.4, 13.7) | 9.5 (6.0, 14.67) | 0.247 |
| IL8 (pg/mL) | 19.3 (16.0, 32.0) | 27.0 (15.9, 33.5) | 0.660 |
| IL10 (pg/mL) | 7.3 (4.6, 10.6) | 10.3 (6.6, 14.2) | 0.129 |
| INF-γ (pg/mL) | 35.1 (23.3, 46.2) | 39.6 (25.2, 54.1) | 0.590 |

**Table S14 Gene set enrichment for gene sets that associate with lung function measured as $TL_{CO}$ % predicted in male patients alone**

| Hallmark gene set | NES | NOM p-val | FDR q-val |
|---|---|---|---|
| **Positive enrichment** | | | |
| OXIDATIVE PHOSPHORYLATION | 2.898 | <0.001 | <0.001 |
| FATTY ACID METABOLISM | 2.444 | <0.001 | 0.000 |
| ADIPOGENESIS | 1.876 | <0.001 | 0.001 |
| PEROXISOME | 1.755 | <0.001 | 0.002 |
| **Negative enrichment** | | | |
| EPITHELIAL MESENCHYMAL TRANSITION | -2.623 | <0.001 | <0.001 |
| UV RESPONSE DN | -2.165 | <0.001 | <0.001 |
| MYC TARGETS V2 | -2.075 | <0.001 | <0.001 |
| INTERFERON GAMMA RESPONSE | -1.971 | <0.001 | 0.001 |
| MYC TARGETS V1 | -1.959 | <0.001 | 0.001 |
| APICAL JUNCTION | -1.890 | <0.001 | 0.001 |
| PROTEIN SECRETION | -1.836 | <0.002 | 0.002 |
| P53 PATHWAY | -1.826 | <0.003 | 0.002 |
| INTERFERON ALPHA RESPONSE | -1.816 | <0.004 | 0.002 |
| TNFA SIGNALING VIA NFKB | -1.791 | <0.005 | 0.002 |
| APOPTOSIS | -1.777 | <0.006 | 0.002 |
| UNFOLDED PROTEIN RESPONSE | -1.773 | <0.007 | 0.001 |
| DNA REPAIR | -1.770 | <0.008 | 0.001 |
| ANDROGEN RESPONSE | -1.740 | <0.009 | 0.002 |
| IL6 JAK STAT3 SIGNALING | -1.685 | 0.002 | 0.004 |
| E2F TARGETS | -1.665 | 0.000 | 0.004 |
| MITOTIC SPINDLE | -1.625 | 0.000 | 0.005 |
| ANGIOGENESIS | -1.622 | 0.006 | 0.005 |
| PI3K AKT MTOR SIGNALING | -1.537 | 0.011 | 0.011 |
| G2M CHECKPOINT | -1.512 | 0.002 | 0.013 |
| IL2 STAT5 SIGNALING | -1.498 | 0.003 | 0.014 |
| ESTROGEN RESPONSE EARLY | -1.398 | 0.015 | 0.035 |
| MYOGENESIS | -1.374 | 0.014 | 0.040 |
| INFLAMMATORY RESPONSE | -1.351 | 0.011 | 0.047 |

**Table S15. Gene set enrichment for Genes correlating with circulating IL1b in male patients at different disease stages**

| MILD COPD patients | | | SEVERE COPD patients | | |
|---|---|---|---|---|---|
| Hallmark gene set | NES | FDR q-val | Hallmark gene set | NES | FDR q-val |
| **Positive enrichment** | | | **Positive enrichment** | | |
| no positive association | | | EPITHELIAL MESENCHYMAL TRANSITION | 2.752 | <0.001 |
| | | | ALLOGRAFT REJECTION | 2.341 | <0.001 |
| | | | TNFA SIGNALING VIA NFKB | 2.332 | <0.001 |
| | | | INTERFERON GAMMA RESPONSE | 2.324 | <0.001 |
| | | | APOPTOSIS | 2.276 | <0.001 |
| | | | MTORC1 SIGNALING | 2.271 | <0.001 |
| | | | IL6 JAK STAT3 SIGNALING | 2.187 | <0.001 |
| | | | INFLAMMATORY RESPONSE | 2.073 | <0.001 |
| | | | KRAS SIGNALING UP | 2.048 | <0.001 |
| **Negative enrichment** | | | COMPLEMENT | 2.001 | <0.001 |
| OXIDATIVE PHOSPHORYLATION | -2.540 | <0.001 | IL2 STAT5 SIGNALING | 1.936 | <0.001 |
| MTORC1 SIGNALING | -2.080 | <0.001 | ANGIOGENESIS | 1.839 | 0.001 |
| UNFOLDED PROTEIN RESPONSE | -1.974 | 0.001 | COAGULATION | 1.736 | 0.002 |
| UV RESPONSE UP | -1.724 | 0.011 | ANDROGEN RESPONSE | 1.720 | 0.003 |
| PEROXISOME | -1.719 | 0.009 | INTERFERON ALPHA RESPONSE | 1.664 | 0.004 |
| FATTY ACID METABOLISM | -1.691 | 0.010 | E2F TARGETS | 1.653 | 0.004 |
| TNFA SIGNALING VIA NFKB | -1.658 | 0.011 | CHOLESTEROL HOMEOSTASIS | 1.627 | 0.005 |
| DNA REPAIR | -1.649 | 0.010 | PROTEIN SECRETION | 1.624 | 0.005 |
| ADIPOGENESIS | -1.578 | 0.016 | G2M CHECKPOINT | 1.492 | 0.017 |
| GLYCOLYSIS | -1.561 | 0.017 | P53 PATHWAY | 1.487 | 0.017 |
| REACTIVE OXYGEN SPECIES PATHWAY | -1.515 | 0.025 | **Negative enrichment** | | |
| MYOGENESIS | -1.473 | 0.033 | MYOGENESIS | -1.786 | 0.003 |
| MYC TARGETS V2 | -1.465 | 0.033 | KRAS SIGNALING DN | -1.570 | 0.029 |
| E2F TARGETS | -1.452 | 0.034 | WNT BETA CATENIN SIGNALING | -1.490 | 0.042 |

**Table S16 Gene set enrichment for Genes correlating with circulating IL10 in male patients at different disease stages**

| MILD COPD | | |
|---|---|---|
| Hallmark gene set | NES | FDR q-val |
| **Positive enrichment** | | |
| No positive gene set associations | | |
| **Negative enrichment** | | |
| INTERFERON GAMMA RESPONSE | -2.877 | <0.001 |
| INTERFERON ALPHA RESPONSE | -2.871 | <0.001 |
| IL6 JAK STAT3 SIGNALING | -2.459 | <0.001 |
| TNFA SIGNALING VIA NFKB | -2.358 | <0.001 |
| EPITHELIAL MESENCHYMAL TRANSITION | -2.192 | <0.001 |
| ALLOGRAFT REJECTION | -2.188 | <0.001 |
| INFLAMMATORY RESPONSE | -2.142 | <0.001 |
| COMPLEMENT | -2.125 | <0.001 |
| APOPTOSIS | -2.033 | <0.001 |
| IL2 STAT5 SIGNALING | -1.989 | 0.001 |
| HEME METABOLISM | -1.838 | 0.002 |
| UV RESPONSE DN | -1.830 | 0.001 |
| PI3K AKT MTOR SIGNALING | -1.750 | 0.002 |
| APICAL JUNCTION | -1.725 | 0.003 |
| ANGIOGENESIS | -1.653 | 0.006 |
| MITOTIC SPINDLE | -1.635 | 0.007 |
| PROTEIN SECRETION | -1.584 | 0.011 |
| ESTROGEN RESPONSE EARLY | -1.540 | 0.016 |
| TGF BETA SIGNALING | -1.487 | 0.023 |
| HYPOXIA | -1.481 | 0.022 |
| COAGULATION | -1.476 | 0.022 |
| MYOGENESIS | -1.473 | 0.022 |
| WNT BETA CATENIN SIGNALING | -1.416 | 0.037 |
| KRAS SIGNALING UP | -1.412 | 0.037 |
| P53 PATHWAY | -1.406 | 0.037 |

| Severe COPD | | |
|---|---|---|
| Hallmark gene set | NES | FDR q-val |
| **Positive enrichment** | | |
| TNFA SIGNALING VIA NFKB | 2.776 | <0.001 |
| EPITHELIAL MESENCHYMAL TRANSITION | 2.657 | <0.001 |
| UV RESPONSE DN | 2.154 | <0.001 |
| APOPTOSIS | 2.091 | <0.001 |
| COAGULATION | 1.838 | 0.002 |
| HYPOXIA | 1.823 | 0.002 |
| TGF BETA SIGNALING | 1.821 | 0.002 |
| ANGIOGENESIS | 1.801 | 0.002 |
| P53 PATHWAY | 1.778 | 0.002 |
| MYC TARGETS V2 | 1.707 | 0.004 |
| INFLAMMATORY RESPONSE | 1.668 | 0.005 |
| MTORC1 SIGNALING | 1.666 | 0.005 |
| IL2 STAT5 SIGNALING | 1.644 | 0.005 |
| IL6 JAK STAT3 SIGNALING | 1.644 | 0.004 |
| PROTEIN SECRETION | 1.615 | 0.006 |
| MYC TARGETS V1 | 1.528 | 0.013 |
| UNFOLDED PROTEIN RESPONSE | 1.492 | 0.018 |
| ANDROGEN RESPONSE | 1.480 | 0.019 |
| MITOTIC SPINDLE | 1.474 | 0.020 |
| KRAS SIGNALING UP | 1.367 | 0.049 |
| COMPLEMENT | 1.362 | 0.049 |
| | | |
| | | |
| **Negative enrichment** | | |
| OXIDATIVE PHOSPHORYLATION | -2.180 | <0.001 |
| FATTY ACID METABOLISM | -1.930 | 0.001 |
| | | |

**Table S17: Genes that form the core enrichment for IL6-STAT signalling with lung function measured as $TL_{CO}$%**

| SYMBOL | RANK IN GENE LIST | RANK METRIC SCORE | RUNNING ES | CORE ENRICHMENT |
|---|---|---|---|---|
| TNFRSF21 | 15816 | -0.33 | 0.01 | Yes |
| FAS | 15709 | -0.31 | -0.05 | Yes |
| IL1R2 | 15712 | -0.31 | -0.02 | Yes |
| OSMR | 15613 | -0.3 | -0.07 | Yes |
| PDGFC | 15373 | -0.27 | -0.09 | Yes |
| CD44 | 14989 | -0.24 | -0.1 | Yes |
| STAT1 | 14825 | -0.23 | -0.16 | Yes |
| STAM2 | 14890 | -0.23 | -0.14 | Yes |
| IRF1 | 14959 | -0.23 | -0.12 | Yes |
| STAT2 | 14788 | -0.22 | -0.18 | Yes |
| TNFRSF12A | 14585 | -0.21 | -0.19 | Yes |
| TYK2 | 14355 | -0.2 | -0.22 | Yes |
| TGFB1 | 14376 | -0.2 | -0.2 | Yes |
| LEPR | 14130 | -0.19 | -0.27 | Yes |
| TNFRSF1B | 14170 | -0.19 | -0.25 | Yes |
| ACVR1B | 14240 | -0.19 | -0.24 | Yes |
| GRB2 | 13889 | -0.18 | -0.29 | Yes |
| CSF3R | 13893 | -0.18 | -0.27 | Yes |
| IL4R | 13629 | -0.17 | -0.31 | Yes |
| MAP3K8 | 13799 | -0.17 | -0.31 | Yes |
| SOCS1 | 13352 | -0.16 | -0.35 | Yes |
| PLA2G2A | 13511 | -0.16 | -0.34 | Yes |
| CD14 | 13535 | -0.16 | -0.33 | Yes |
| TLR2 | 13073 | -0.15 | -0.41 | Yes |
| CXCL3 | 13122 | -0.15 | -0.4 | Yes |
| IL17RA | 13210 | -0.15 | -0.39 | Yes |
| CXCL9 | 13240 | -0.15 | -0.38 | Yes |
| PTPN2 | 13251 | -0.15 | -0.36 | Yes |
| IL10RB | 12716 | -0.14 | -0.44 | Yes |
| PTPN1 | 12782 | -0.14 | -0.43 | Yes |
| IRF9 | 12862 | -0.14 | -0.42 | Yes |
| IFNGR2 | 12404 | -0.13 | -0.43 | Yes |
| IL2RG | 12064 | -0.12 | -0.44 | Yes |
| ITGB3 | 12285 | -0.12 | -0.44 | Yes |
| PTPN11 | 11699 | -0.11 | -0.45 | Yes |
| CSF2RB | 11756 | -0.11 | -0.44 | Yes |
| CXCL10 | 11885 | -0.11 | -0.44 | Yes |
| PF4 | 11276 | -0.1 | -0.45 | Yes |
| STAT3 | 11292 | -0.1 | -0.44 | Yes |
| ITGA4 | 11349 | -0.1 | -0.44 | Yes |

**Table S18: Gene sets enrichment for genes that associated with myc in the muscle of patients with COPD**

| NAME | NES | NOM p-val | FDR q-val |
|---|---|---|---|
| **Positive enrichment** | | | |
| EPITHELIAL MESENCHYMAL TRANSITION | 3.210 | <0.001 | <0.001 |
| TNFA SIGNALING VIA NFKB | 3.177 | <0.001 | <0.001 |
| MYC TARGETS V2 | 2.538 | <0.001 | <0.001 |
| APOPTOSIS | 2.534 | <0.001 | <0.001 |
| UNFOLDED PROTEIN RESPONSE | 2.395 | <0.001 | <0.001 |
| IL2 STAT5 SIGNALING | 2.331 | <0.001 | <0.001 |
| MTORC1 SIGNALING | 2.319 | <0.001 | <0.001 |
| ANGIOGENESIS | 2.256 | <0.001 | <0.001 |
| MYC TARGETS V1 | 2.255 | <0.001 | <0.001 |
| INFLAMMATORY RESPONSE | 2.243 | <0.001 | <0.001 |
| IL6 JAK STAT3 SIGNALING | 2.227 | <0.001 | <0.001 |
| INTERFERON GAMMA RESPONSE | 2.139 | <0.001 | <0.001 |
| P53 PATHWAY | 2.084 | <0.001 | <0.001 |
| COAGULATION | 2.046 | <0.001 | <0.001 |
| COMPLEMENT | 2.009 | <0.001 | <0.001 |
| UV RESPONSE DN | 1.977 | <0.001 | <0.001 |
| CHOLESTEROL HOMEOSTASIS | 1.931 | <0.001 | <0.001 |
| UV RESPONSE UP | 1.886 | <0.001 | 0.001 |
| ANDROGEN RESPONSE | 1.849 | <0.001 | 0.001 |
| KRAS SIGNALING UP | 1.843 | <0.001 | 0.001 |
| HYPOXIA | 1.840 | <0.001 | 0.001 |
| APICAL JUNCTION | 1.830 | <0.001 | 0.001 |
| TGF BETA SIGNALING | 1.826 | <0.001 | 0.001 |
| ALLOGRAFT REJECTION | 1.794 | <0.001 | 0.001 |
| E2F TARGETS | 1.748 | <0.001 | 0.001 |
| INTERFERON ALPHA RESPONSE | 1.727 | <0.001 | 0.002 |
| G2M CHECKPOINT | 1.707 | <0.001 | 0.002 |
| XENOBIOTIC METABOLISM | 1.578 | <0.001 | 0.008 |
| GLYCOLYSIS | 1.539 | 0.004 | 0.011 |
| PI3K AKT MTOR SIGNALING | 1.427 | 0.023 | 0.027 |
| MITOTIC SPINDLE | 1.386 | 0.015 | 0.037 |
| **Negative enrichment** | | | |
| OXIDATIVE PHOSPHORYLATION | -2.076 | <0.001 | <0.001 |
| ADIPOGENESIS | -1.854 | <0.001 | <0.001 |
| BILE ACID METABOLISM | -1.832 | <0.001 | <0.001 |
| FATTY ACID METABOLISM | -1.795 | <0.001 | <0.001 |
| HEME METABOLISM | -1.610 | <0.001 | 0.006 |
| KRAS SIGNALING DN | -1.468 | 0.006 | 0.022 |
| MYOGENESIS | -1.392 | 0.006 | 0.038 |

**Table S19: Gene set Enrichment analysis in the lung of patients with severe compared to mild COPD where all patients have emphysema**

| Hallmark gene set | NES | FDR q-val |
|---|---|---|
| **Positive enrichment** | | |
| ALLOGRAFT REJECTION | 2.44 | <0.001 |
| E2F TARGETS | 2.35 | <0.001 |
| MYC TARGETS V2 | 2.27 | <0.001 |
| IL6 JAK STAT3 SIGNALING | 2.16 | <0.001 |
| INTERFERON GAMMA RESPONSE | 2.04 | <0.001 |
| G2M CHECKPOINT | 2.00 | <0.001 |
| INFLAMMATORY RESPONSE | 2.00 | <0.001 |
| MTORC1 SIGNALING | 1.87 | 0.001 |
| COMPLEMENT | 1.77 | 0.003 |
| KRAS SIGNALING UP | 1.69 | 0.005 |
| OXIDATIVE PHOSPHORYLATION | 1.68 | 0.005 |
| UNFOLDED PROTEIN RESPONSE | 1.66 | 0.006 |
| GLYCOLYSIS | 1.62 | 0.008 |
| EMT | 1.58 | 0.011 |
| INTERFERON ALPHA RESPONSE | 1.56 | 0.012 |
| COAGULATION | 1.55 | 0.012 |
| XENOBIOTIC METABOLISM | 1.55 | 0.013 |
| REACTIVE OXYGEN SPECIES PATHWAY | 1.45 | 0.025 |
| ESTROGEN RESPONSE LATE | 1.45 | 0.025 |
| IL2 STAT5 SIGNALING | 1.42 | 0.029 |
| SPERMATOGENESIS | 1.40 | 0.034 |
| PI3K AKT MTOR SIGNALING | 1.39 | 0.034 |
| MYC TARGETS V1 | 1.35 | 0.045 |
| **Negative enrichment** | | |
| UV RESPONSE DN | -2.18 | <0.001 |
| TGF BETA SIGNALING | -2.05 | <0.001 |
| MYOGENESIS | -1.85 | 0.001 |
| HEDGEHOG SIGNALING | -1.78 | 0.003 |
| APICAL JUNCTION | -1.77 | 0.003 |
| WNT BETA CATENIN SIGNALING | -1.75 | 0.003 |
| TNFA SIGNALING VIA NFKB | -1.55 | 0.019 |
| ESTROGEN RESPONSE EARLY | -1.53 | 0.020 |
| APOPTOSIS | -1.50 | 0.024 |
| PROTEIN SECRETION | -1.47 | 0.028 |
| NOTCH SIGNALING | -1.46 | 0.031 |
| HYPOXIA | -1.42 | 0.040 |
