# Supplementary figures and images for: Variability in sensitivity to inflammation in muscle and lung of patients with COPD may underlie susceptibility to lung function decline
Source: Thorax. 2025 Apr 16;80(8):e221901. doi: 10.1136/thorax-2024-221901 (PMC12322413; doi:10.1136/thorax-2024-221901)

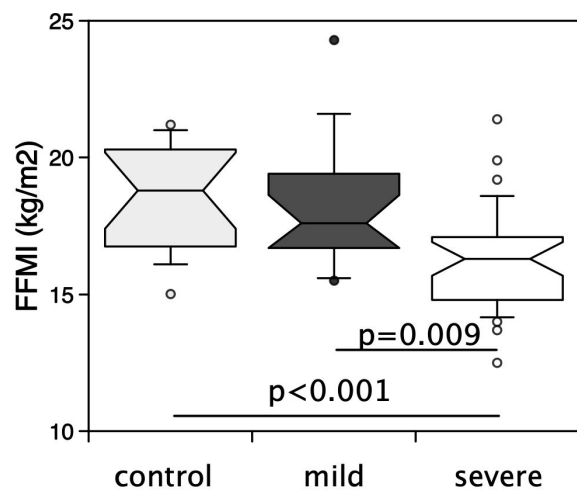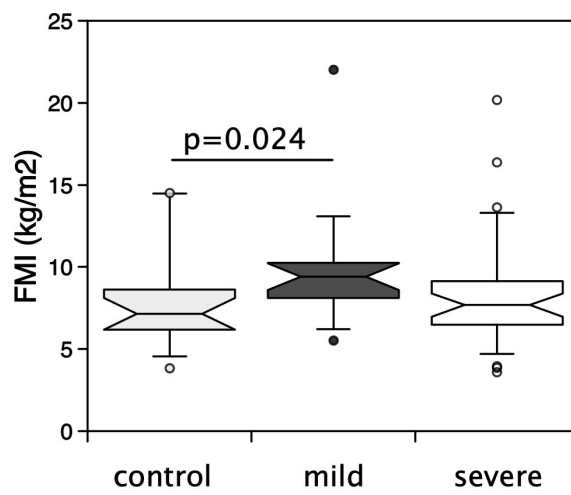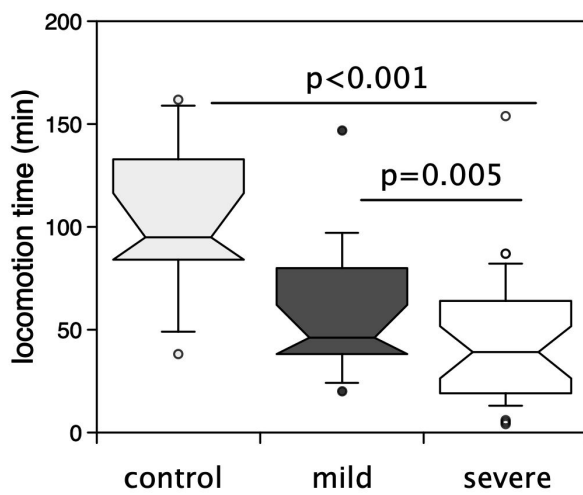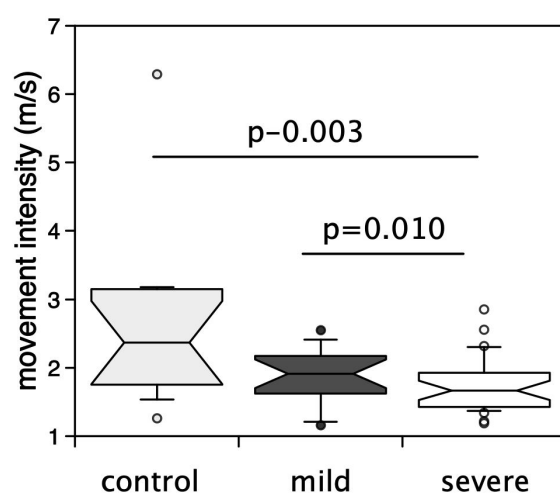

males only

Figure S1

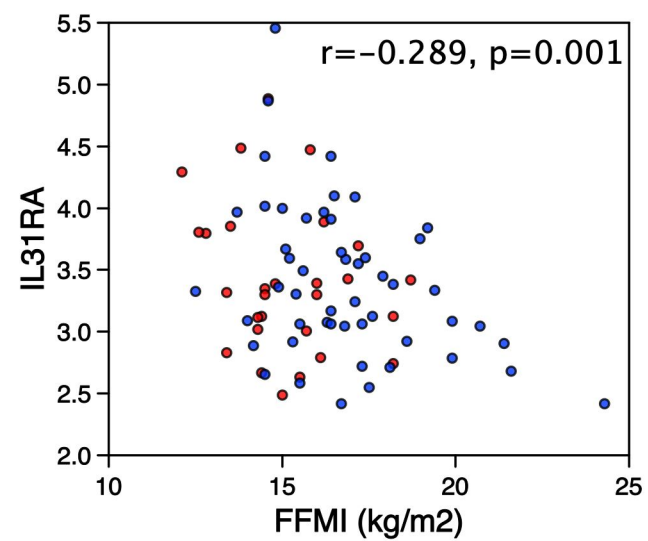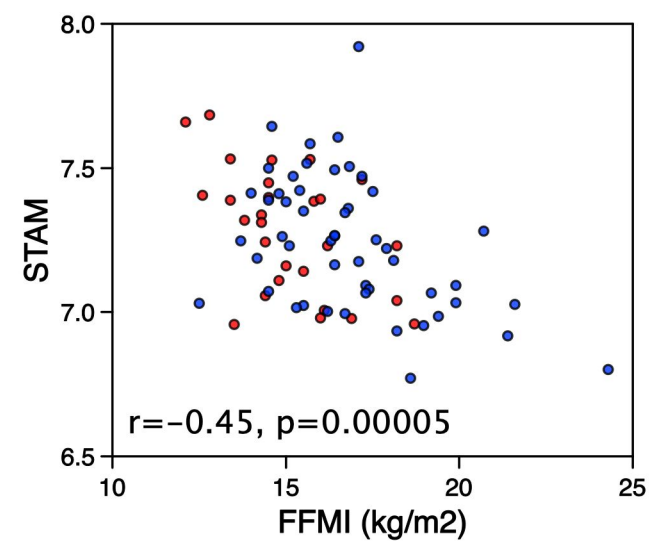

Figure S2

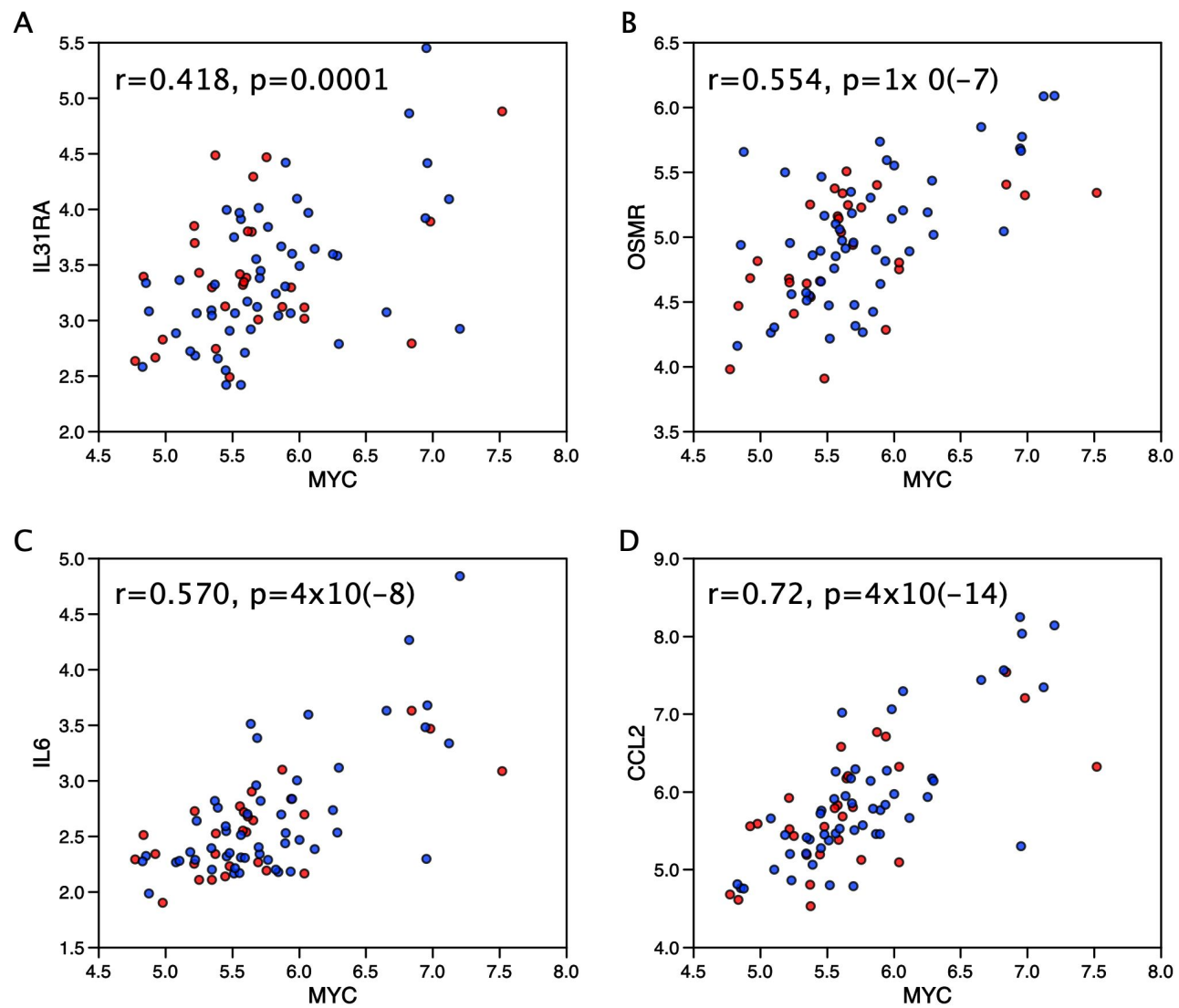

Figure S3

Supplement: online supplemental file 3 [file thorax-80-8-s003.pdf]
