# Supplementary material for: Variability in sensitivity to inflammation in muscle and lung of patients with COPD may underlie susceptibility to lung function decline
Source: Thorax. 2025 Apr 16;80(8):e221901. doi: 10.1136/thorax-2024-221901 (PMC12322413; doi:10.1136/thorax-2024-221901)
Supplement: online supplemental file 4 [file thorax-80-8-s004.pptx]

## Slide 1
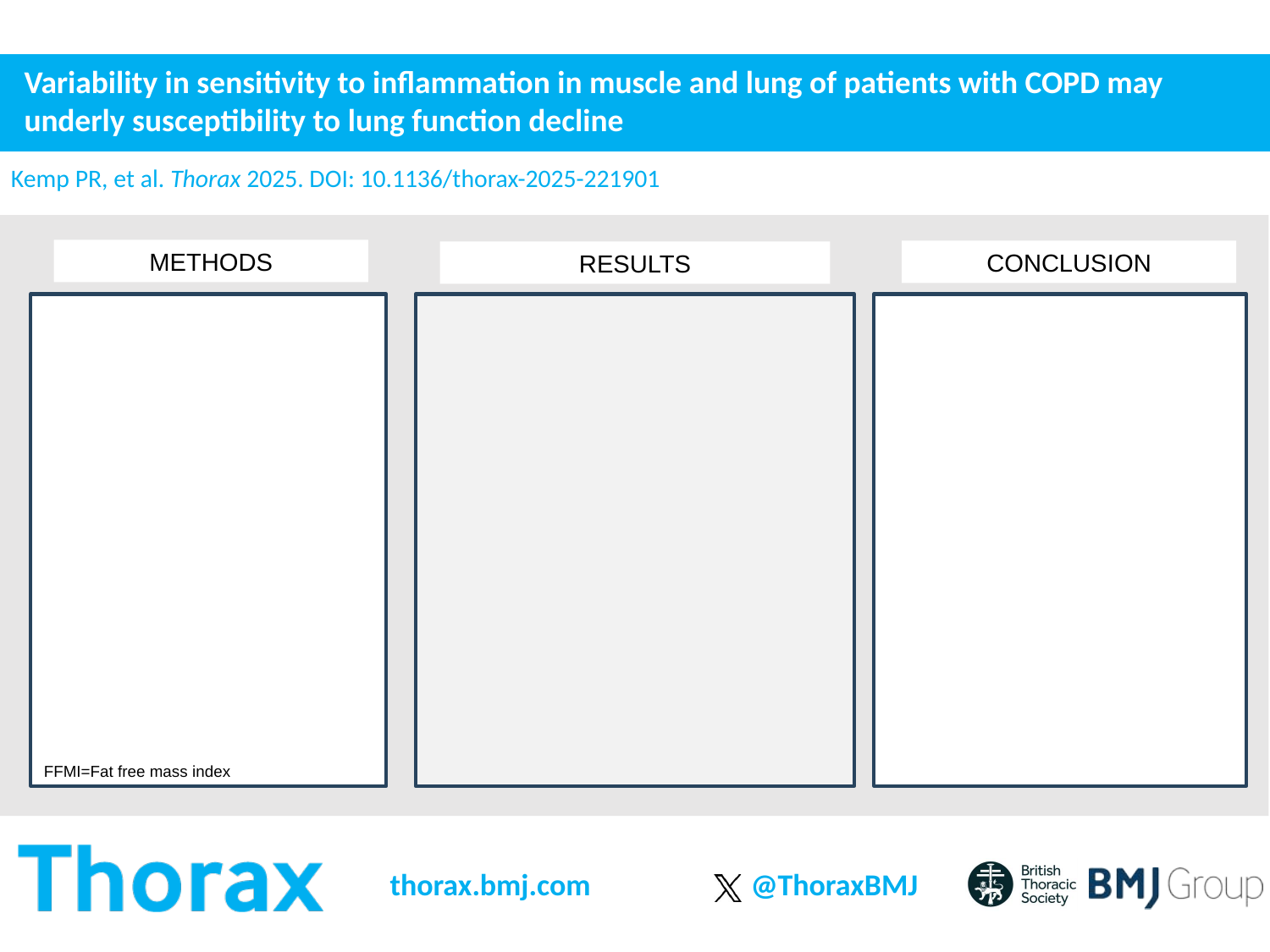

Variability in sensitivity to inflammation in muscle and lung of patients with COPD may underly susceptibility to lung function decline
Kemp PR, et al. Thorax 2025. DOI: 10.1136/thorax-2025-221901
METHODS
CONCLUSION
RESULTS
Manuscript Title
FFMI=Fat free mass index
thorax.bmj.com @ThoraxBMJ
